# Cloud-Based Commensality: Enjoy the Company of Co-diners Without Social Facilitation of Eating

**DOI:** 10.3389/fpsyg.2021.758966

**Published:** 2021-11-12

**Authors:** Chujun Wang, Yubin Peng, Linbo Qiu, Xiaoang Wan

**Affiliations:** Department of Psychology, Tsinghua University, Beijing, China

**Keywords:** digital commensality, Mukbang watching, videoconferencing, loneliness, social facilitation

## Abstract

Previous research has associated frequently enforced solo dining with negative consequences on psychological well-being, but the problem of having to eat alone may be solved by seeking mealtime companions in the digital space by watching an eating broadcast (i.e., Mukbang) or videoconferencing with others (i.e., cloud-based commensality). We conducted the present study to compare the consequences of Mukbang-based, cloud-based, and in-person commensality. Ninety-five healthy Chinese young adults were instructed to rate images of eating scenarios and foods. The results revealed that they expected loneliness to be reduced by Mukbang-based or in-person commensality, but they were also aware of the risks of enhancing food intake and/or being shifted toward less healthy food choices in these two scenarios. By contrast, the participants expected cloud-based commensality to provide the benefits of reducing loneliness without the health-compromising risks of increasing food intake or unhealthy eating. Collectively, these findings suggest the beliefs of the participants that cloud-based commensality can provide an “alone but together” context to balance the need for social interactions with the strategic avoidance of a social context facilitating unhealthy eating. The findings also provide some novel insights into how the application of technologies for eating behavior can be used to integrate social factors and food pleasure, and shed light on the promising future of cloud-based commensality as a combination of the strengths of solitary and commensal eating.

## Introduction

As social creatures, we spend approximately 80% of our waking hours with the company of others (Kahneman et al., 2004) and often find hedonic activities more enjoyable when engaging with others (Ragunathan and Corfman, 2006), such as eating together (i.e., commensality). However, eating alone has become a rising trend due to many socioeconomic factors, such as the increases in single-person households, the growth in the aging population, and the pressures of busy lifestyles (Klinenberg, 2013; Spence, 2017). The prevalence of eating alone does make a person miss out on the social benefits of commensality, which may be associated with the increases in the feeling of unhappiness (Yiengprugsawan et al., 2015) or the decreases in diet quality (Chae et al., 2018), as well as foster loneliness and the perception of social isolation (Takeda and Melby, 2017). By contrast, eating together not only provides opportunities for social interactions and social bonding (Sobal and Nelson, 2003) but also can make palatable foods taste even better (Hetherington et al., 2006).

Even though eating with others may expose an individual to harmful social norms and thus increase one’s food intake (Cruwys et al., 2015), these risks are outweighed by the potential benefits to one’s physical, social, and emotional well-being (Fulkerson et al., 2014). When the circumstances make it difficult or even impossible to eat with others, advances in technologies allow a solo diner to seek mealtime companions in the digital space, resulting in a new form of commensality between remote co-diners (Grevet et al., 2012). Spence et al. (2019) used the term “digital commensality” to describe scenarios that enable a solo diner to have the feeling of eating with others *via* digital technologies, such as watching Mukbang or videoconferencing with other diners while eating alone in reality.

Specifically, Mukbang refers to online broadcasts in which a host or hostess eats large portions of energy-dense foods on camera while interacting with online viewers (Kircaburun et al., 2020). Many viewers choose to watch Mukbang for satisfying psychological and/or social needs, such as obtaining vicarious satisfaction of a desire to eat through visual and audio stimulation (Choe, 2019), or reducing social isolation, especially during the recent COVID-19 crisis (Kang et al., 2021). It is worth noting that watching Mukbang without eating is vicarious enough to fulfill the need of viewers for entertainment and sensory satisfaction (Choe, 2019), and they can choose whether to eat at the same time while watching Mukbang. That being said, many viewers choose to watch Mukbang while they are eating alone in reality, resulting in the emergence of Mukbang-based commensality (Spence et al., 2019; Anjani et al., 2020). By contrast, a solo diner can also choose to conduct both visual and audio interactions with remote diners *via* videoconferencing tools. This type of commensality is referred to as Skeating (i.e., a term coined from “Skyping” and “eating”) by Spence et al. (2019), although it is widely known as “cloud-based union dinner” in the global Chinese community due to the role of cloud computing in videoconferencing technologies (Ma, 2021). Considering the importance of dissociating this term with any specific software, we chose to use the term “cloud-based commensality” in this study.

Many studies have demonstrated that videoconferencing allows for efficient interactions in long-distance education (Saw et al., 2008) and psychotherapy (Simpson, 2009), and previous research on elderly nursing home residents has shown that videoconferencing can alleviate feelings of loneliness (Hsiu-Hsin et al., 2010). Indeed, both Mukbang-based and cloud-based commensality can elicit feelings of eating with others (Spence et al., 2019), as both of them can elicit perceptions of social presence (Lowden and Hostetter, 2012; Choe, 2019). That being said, to the best of our knowledge, it remains unclear how Mukbang-based and cloud-based commensality differ from each other, or from in-person commensality, in terms of the consequences on one’s emotional state, food intake, and food choices. Therefore, we conducted the present study to address this issue.

In terms of the restriction of the availability of some complex social information (e.g., nonverbal cues; Straus and McGrath, 1994), and the risks of distracting a diner with screen-based devices (Marsh et al., 2013), digital interactions cannot exactly substitute for in-person communication. That said, we still cannot ignore the advantages of digital commensality. For one thing, considering the health risk and loneliness caused by lockdown during the recent COVID-19 crisis (Holmes et al., 2020), cloud-based commensality *via* videoconferencing tools has been identified as a safe and efficient alternative to social gatherings and loneliness regulation (Ceccaldi et al., 2020; Spence et al., 2021). For another, in-person and cloud-based commensality could both elicit the feeling of being accompanied by family or friends (Cruwys et al., 2015; Ceccaldi et al., 2020). By contrast, Mukbang-based commensality may be less effective in reducing loneliness, as the mealtime companions it provides are a broadcast host or hostess and other online viewers (Kircaburun et al., 2020). However, Mukbang-based commensality may satisfy the need of a person for privacy when eating (Donnar, 2017), whereas in-person commensality inevitably transforms eating from a private behavior to a public act being observed (Fischler, 2011). Therefore, we developed the first hypothesis as follows.

H1: In-person and cloud-based commensalities offer similar benefits of promoting social connectedness, whereas Mukbang-based commensality also has such social benefits but is less effective in reducing loneliness in comparison with in-person and cloud-based commensalities.

Second, we expected to find similarities between in-person and Mukbang-based commensality in terms of the influence on the food intake of an individual (Cruwys et al., 2015; Donnar, 2017). A recent study has shown that in-person commensality can elicit more pleasantness from consuming unhealthy foods than solo dining (Huang et al., under review). Compared with traditional, in-person commensality, the risks of enhancing one’s food intake in cloud-based commensality may be reduced or even minimized, as the eating behavior of a solo diner is not very likely to be influenced by a remote co-diner who is eating in a different context (Hermans et al., 2012). Moreover, although both Mukbang- and cloud-based commensalities involve the remote co-diners in eating in different contexts (Hermans et al., 2012), Mukbang viewers are always repeatedly exposed to excessive consumption of energy-dense foods of the host or hostess (Strand and Gustafsson, 2020), which can enhance the perceived appropriateness of unhealthy eating (Poor et al., 2013). Mukbang-based commensality has been proved to be associated with a number of health risks, such as increasing food intake due to social comparison or mimicry, or underestimating the harmfulness of overindulgence (Donnar, 2017; Strand and Gustafsson, 2020). Therefore, we developed the following hypothesis.

H2: Both in-person and Mukbang-based commensalities could potentially increase the probabilities of choosing unhealthy foods compared with solo dining; whereas cloud-based commensality might not have such detrimental effects.

## Materials and Methods

This study was conducted in accordance with the ethical standards laid down in the Declaration of Helsinki, approved by the Institution Review Board of a major research university in Beijing, China, and was conducted from December 2020 to June 2021.

### Participants and Recruitment

We conducted an *a priori* power analysis for sample size estimation using the G^∗^Power software, using a medium effect size *f* of 0.25 (Cohen, 2013), an alpha of 0.05, and a power of 0.95 for the analysis. The projected sample size was 43 at the minimum, calculated on the basis of a one-way repeated-measure ANOVA with three factors and a 4 × 2 repeated-measure ANOVA. Ninety-five healthy Chinese young adults (mean age = 21.3 ± 2.5 years, ranging from 18 to 29 years; 43 males and 52 females) were recruited to take part in this study. They were undergraduate or graduate students of a major research university in Beijing and were recruited through posts on social media or on-campus flyers. They all reported of having normal or corrected-to-normal vision without color blindness, and none of them reported having symptoms of flu or fever in the recent period of time. Each participant had signed informed consent electronically before the study and received 20 Chinese Yuan (equal to approximately 3 USD when the study was conducted) after completion.

### Apparatus and Stimuli

**FIGURE 1:**
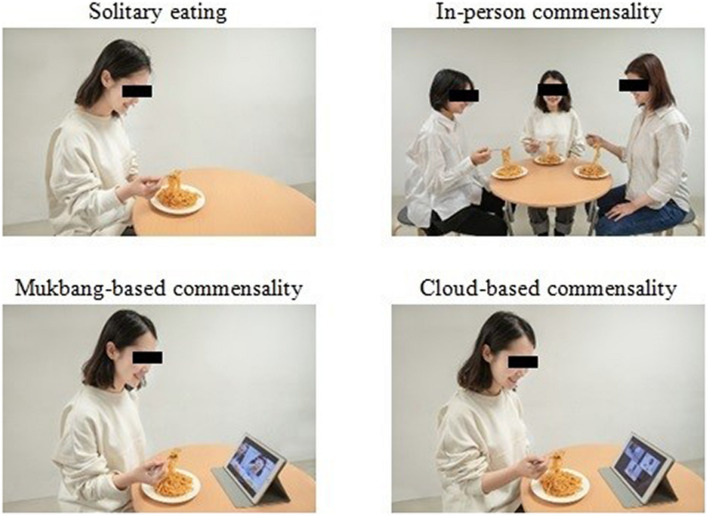
Scenario photos of female diners. Note that we blocked the faces of the diners with black bars to protect their privacy, but the photos were presented to the participants without the black bars in the study.

In order to show different eating scenarios to the participants, we took different versions of photos of a Chinese young adult who was eating Asian noodles. Considering that we planned to ask the participants to imagine being in this scenario during the study, we took photos of female and male diners separately and showed them to the female and male participants, respectively. The photos of the female diners presented to the female participants are shown in Figure 1 as illustrative examples. In each photo, a young woman was eating when alone, when having the company of two co-diners (of the same sex) using individual plates, when watching Mukbang *via* a tablet computer, or when videoconferencing with two co-diners (of the same sex) *via* a tablet computer. When each photo was presented on the computer screen (720 pixels wide × 480 pixels high), a sentence was shown above the image to ensure the understanding of the scenario, including “you are eating alone,” “you are eating with your friends, and everyone has his or her own plate of food,” “you are eating while watching an eating broadcast,” or “you are eating while videoconferencing with your eating friends.”

We also selected eight food photos from the Full4Health Image Collection (the food images numbered as 51, 75, 76, 77, 125, 233, 317, and 333) created by Charbonnier et al. (2016). Each photo presented a kind of food that is commonly seen in our local diets against a gray background. Half of these foods were more energy-dense (313 vs. 71 kcal/100 g) and fattier (*M* = 13.5 vs. 1.9 g/100 g) than the rest of the foods, both *t*s > 3.46, *p*s < 0.02. Therefore, these foods were sorted into two groups, including a group of unhealthy foods (i.e., pizza, cheeseburgers, cakes, and biscuits) and a group of healthy foods (i.e., vegetable salads, cod filet, fruit salads, and slices of apple). When each food photo was shown on the computer screen (631 pixels wide × 422 pixels high), a label of food name (consisting of two or three Chinese characters) and a label of eating scenario (consisting of five Chinese characters) were shown above and below the photo, respectively.

### Design and Procedure

The participants were asked to visit a psychology lab to complete a survey online at www.qualtrics.com, and they were asked to fast at least 1 h before the study. This study consisted of a phase of scenario ratings and a phase of food ratings. As for scenario ratings, we used a within-participants design by presenting the photos of three forms of commensality in a random order, one at a time. When viewing each photo, the participants were instructed to imagine being in this scenario. Subsequently, they were asked to perform the following tasks. First and second, they were asked to rate the relative changes on loneliness and on food intake caused by this scenario compared to solitary eating, all on seven-point scales (1 = reduced, 4 = unchanged, and 7 = enhanced). Similar to Meng et al. (2020), we asked our participants to rate three aspects of their own loneliness (I feel left out, I feel isolated, and I lack companionship), while imagining being in the scenario, and used the average responses of these three items as a single measure in data analyses. Third, our participants were asked to rate the extent to which this scenario was public or private on a seven-point scale (with 1 = very private, 4 = neutral, and 7 = very public). Fourth, they were also asked to indicate how frequently this type of eating occurred in their life (never, occasionally, sometimes, or daily).

As for food ratings, we used a 4 (Scenario: solitary eating, in-person commensality, Mukbang-based commensality, or cloud-based commensality) × 2 (Food type: unhealthy or healthy foods) within-participants design. Each participant completed four blocks of eight trials each, and a scenario was randomly chosen to use for each block. At the beginning of each block, a scenario photo was presented, and the participants were instructed to imagine being in this scenario. After that, eight food photos were presented in a random order, one at a time. While viewing each food image, they were asked to indicate the probability of choosing this food (ranging from very unlikely to very likely) and the expected pleasantness of eating this food (ranging from not pleasant at all to very pleasant), both on seven-point scales.

At the end of the study, we asked the participants to provide ratings of familiarity, pleasantness, palatability, healthiness, and energy-density for each food, all on seven-point scales with larger numbers indicating the increased intensity of the attribute being rated. The data of these ratings were collected for the purpose of manipulation check, so the images were presented without any labels.

## Results

### Manipulation Check

First, we analyzed the ratings of unhealthy and healthy foods without any labels. Compared with healthy foods, unhealthy foods were considered as being more familiar (*M*_*unhealthy*_ = 6.3 ± 0.7 vs. *M*_*healthy*_ = 5.5 ± 1), more pleasant (*M*_*unhealthy*_ = 5.4 ± 0.8 vs. *M*_*healthy*_ = 4.6 ± 1), more palatable (*M*_*unhealthy*_ = 5.4 ± 0.8 vs. *M*_*healthy*_ = 4.6 ± 0.9), more energy-dense (*M*_*unhealthy*_ = 5.9 ± 0.6 vs. *M*_*healthy*_ = 2.8 ± 0.8), but less healthy (*M*_*unhealthy*_ = 3.1 ± 0.8 vs. *M*_*healthy*_ = 6.1 ± 0.5), all *F*s > 35.82, *p*s < 0.001, η_*p*_^2^s > 0.27. These results indicated that our manipulation of food healthiness was valid. Moreover, it should be noted that the familiarity, pleasantness, and palatability scores of both unhealthy and healthy foods were higher than the middle point of the seven-point scales, all *t*s > 6.09, *p*s < 0.001, Cohen’s *d*s > 0.62. These results revealed that both unhealthy and healthy foods used in this study were pleasant and palatable, and our participants were quite familiar with them.

### Scenarios Ratings

**FIGURE 2:**
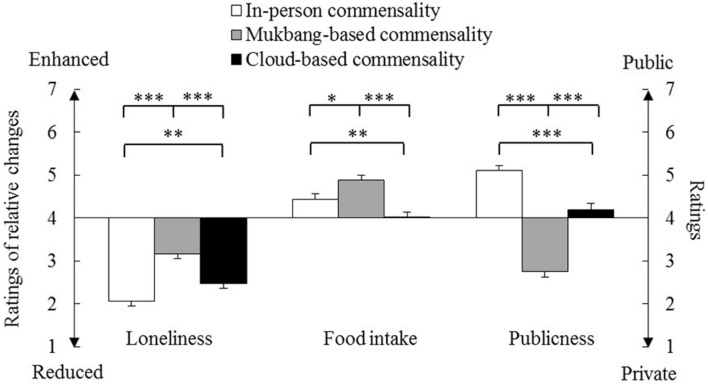
Mean scores of scenario ratings. The error bars show the standard errors of the means. ^∗^*p* < 0.05, ^∗∗^*p* < 0.01, and ^∗∗∗^*p* < 0.001.

The mean scores of scenario ratings are shown in Figure 2. We conducted one-way repeated-measure ANOVAs on these data. As summarized in Table 1, the results revealed a significant main effect of Scenario on all three measures. We performed pairwise comparisons with Bonferroni’s correction and one-sample *t*-tests against the middle points of the seven-point scales (i.e., 4) that represented “unchanged” or “neutral” (also see Table 1). Note that we used Bonferroni’s correction for this and following comparisons, and only reported the *p-*values after such correction.

**TABLE 1:** The results of ANOVA, pairwise comparisons, and one-sample *t*-tests performed on the data of scenario ratings.

Measure	Loneliness		Food intake		Publicness	
(ANOVA)	*F*(2,188)	η_*p*_^2^	*F*(2,188)	η_*p*_^2^	*F*(2,188)	η_*p*_^2^
Main effect of Scenario	36.95***	0.28	17.91***	0.16	106.80***	0.53
(Pairwise comparisons with Bonferroni correction)	*t*(94)	Cohen’s *d*	*t*(94)	Cohen’s *d*	*t*(94)	Cohen’s *d*
In-person vs. Mukbang	7.67***	0.79	2.97*	0.31	14.39***	1.48
In-person vs. Cloud	3.48**	0.36	3.06**	0.31	5.87***	0.60
Mukbang vs. Cloud	5.44***	0.56	5.93***	0.61	8.56***	0.88
(One-sample *t-*tests vs. the middle point)	*t*(94)	Cohen’s *d*	*t*(94)	Cohen’s *d*	*t*(94)	Cohen’s *d*
In-person	17.66***	1.81	3.62**	0.37	9.20***	0.94
Mukbang	6.54***	0.67	9.07***	0.93	9.60***	0.99
Cloud-based	13.99***	1.44	0.27	–	1.33	–

*p < 0.05, **p < 0.01, and ***p < 0.001.

As for the rating of relative changes on loneliness, in-person and Mukbang-based commensality received the lowest and the highest scores, respectively. All three scenarios received scores lower than the middle point. These results revealed that all three forms of commensality were expected to reduce loneliness, and cloud-based commensality was expected to be more effective than Mukbang-based commensality.

As for the ratings of relative changes on food intake, Mukbang-based and cloud-based commensalities received the highest and the lowest scores, respectively. Both in-person and Mukbang-based commensalities received scores higher than the middle point; whereas no such difference was found for cloud-based commensality. These results revealed that both in-person and Mukbang-based commensalities were expected to significantly increase food intake compared with solitary eating.

As for the ratings of publicness, in-person and Mukbang-based commensalities received the highest and the lowest scores. In-person and Mukbang-based commensalities received scores significantly higher and lower than the middle point, respectively, whereas no such effect was found for cloud-based commensality. These results revealed that in-person and Mukbang-based commensalities might be considered as public and private situations, respectively.

Moreover, the percentages of the participants who reported that each type of commensality never, occasionally, sometimes, or daily occurred in their life are shown in Table 2.

**TABLE 2:** The percentages of the participants who reported that each type of eating scenario never, occasionally, sometimes, and daily occurred in their life.

Eating scenario	Never	Occasionally	Sometimes	Daily
In-person commensality	1%	23%	47%	29%
Mukbang-based commensality	42%	28%	19%	11%
Cloud-based commensality	68%	26%	6%	0%

### Food Ratings

**FIGURE 3:**
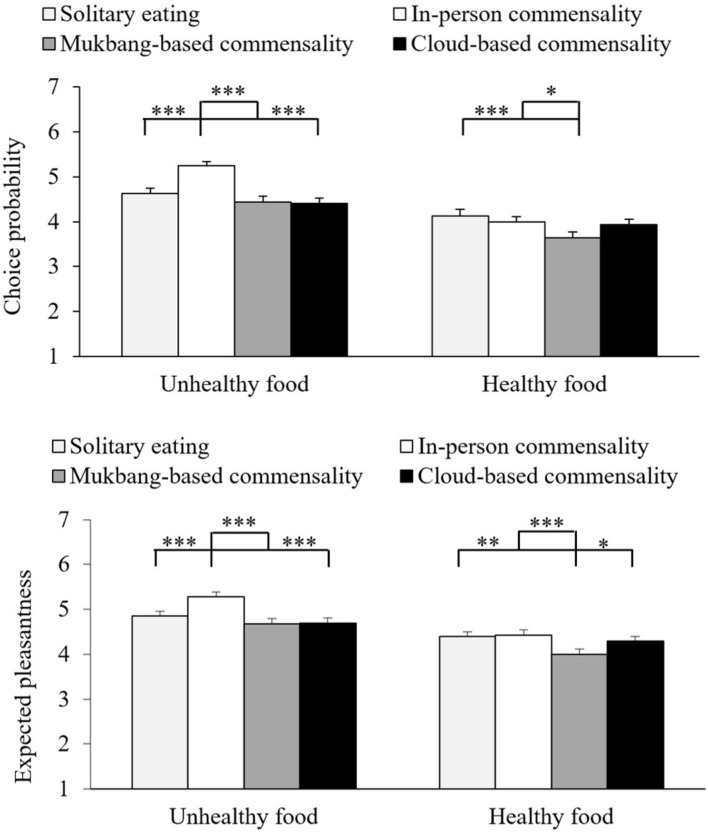
Mean scores of food ratings. The error bars show the standard errors of the means. ^∗^*p* < 0.05, ^∗∗^*p* < 0.01, and ^∗∗∗^*p* < 0.001.

The mean scores of food ratings are shown in Figure 3. We performed 4 (Scenario: solitary eating, in-person commensality, Mukbang-based commensality, or cloud-based commensality) × 2 (Food type: unhealthy or healthy foods) repeated-measure ANOVAs on these data. As summarized in Table 3, the results revealed a significant main effect of Food Type on both measures. These results indicated that the participants were more likely to choose unhealthy foods (*M*_*unhealthy*_ = 4.7 ± 0.8 vs. *M*_*healthy*_ = 3.9 ± 1) and expect more pleasantness from eating unhealthy foods (*M*_*unhealthy*_ = 4.9 ± 0.8 vs. *M*_*healthy*_ = 4.3 ± 0.9) compared with healthy foods. The results also revealed a significant main effect of Scenario on both measures, but they were both qualified by significant interaction terms. We then conducted simple-effects analyses to interpret the significant interaction terms.

**TABLE 3:** The results of ANOVA and pairwise comparisons performed on the data of food ratings.

Measure		Choice probability		Expected pleasantness	
(ANOVA)		*F*(3,282)	η_*p*_^2^	*F*(3,282)	η_*p*_^2^
Main effect of scenario		14.61***	0.14	15.77***	0.14
		*F*(1,94)	η_*p*_^2^	*F*(1,94)	η_*p*_^2^
Main effect of food type		37.27***	0.28	26.17***	0.22
		*F*(3,282)	η_*p*_^2^	*F*(3,282)	η_*p*_^2^
Interaction term		13.61***	0.13	7.06***	0.07
(Pairwise comparisons)	Foods	*t*(94)	Cohen’s *d*	*t*(94)	Cohen’s *d*
Solitary vs. In-person	Unhealthy	5.01***	0.51	4.64***	0.48
	Healthy	1.15	–	0.57	–
Solitary vs. Mukbang	Unhealthy	1.78	–	1.84	–
	Healthy	4.18***	0.43	3.90***	0.40
Solitary vs. Cloud	Unhealthy	1.75	–	1.67	–
	Healthy	1.80	–	1.19	–
In-person vs. Mukbang	Unhealthy	6.82***	0.70	5.61***	0.58
	Healthy	2.84*	0.29	3.96***	0.41
In-person vs. Cloud	Unhealthy	7.79***	0.80	6.56***	0.67
	Healthy	0.55	–	1.59	–
Mukbang vs. Cloud	Unhealthy	0.24	–	0.15	–
	Healthy	2.42	–	2.94*	0.30

*p < 0.05 and ***p < 0.001.

As for the ratings of choice probability, subsequent one-way repeated-measure ANOVAs revealed a significant main effect of Scenario for both unhealthy foods, *F*(3,282) = 22.382, *p* < 0.001, η_*p*_^2^ = 0.19, and healthy foods, *F*(3,282) = 6.22, *p* < 0.001, η_*p*_^2^ = 0.06. As also summarized in Table 2, pairwise comparisons revealed that in-person commensality elicited the highest probability of choosing unhealthy foods. Mukbang-based commensality elicited a lower probability of choosing healthy foods than solitary eating or in-person commensality, whereas none of other pairwise comparisons were significant.

As for the ratings of expected pleasantness, subsequent one-way repeated-measure ANOVAs revealed a significant main effect of Scenario for both unhealthy foods, *F*(3,282) = 16.56, *p* < 0.001, η_*p*_^2^ = 0.15, and healthy foods, *F*(3,282) = 8.87, *p* < 0.001, η_*p*_^2^ = 0.09. As summarized in Table 2, pairwise comparisons revealed that in-person commensality was expected to elicit the highest level of pleasantness from eating unhealthy foods, whereas Mukbang-based commensality was expected to elicit the lowest level of pleasantness from eating healthy foods. None of the other pairwise comparisons were significant.

**FIGURE 4:**
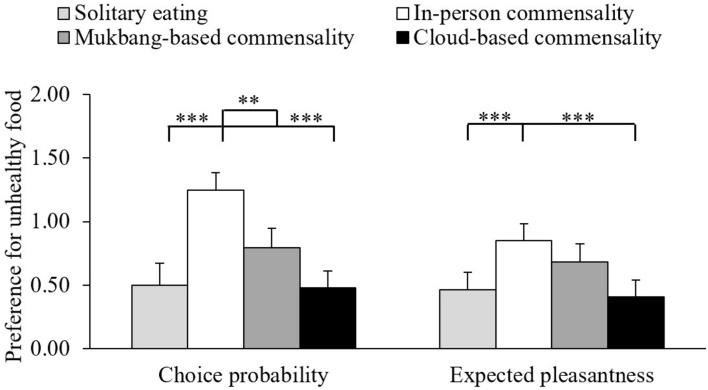
The mean scores of preferences for unhealthy foods (over healthy foods) in different scenarios. The error bars show the standard errors of the means. ^∗∗^*p* < 0.01, and ^∗∗∗^*p* < 0.001.

Furthermore, we indexed the preference for unhealthy foods (over healthy foods) by subtracting the scores of healthy foods from those of unhealthy foods for each scenario separately (see Figure 4). Based on the results of choice probability, our participants showed stronger preference for unhealthy foods when imagining engaging in in-person commensality than solitary eating, *t*(94) = 4.95, *p* < 0.001, Cohen’s *d* = 0.51, cloud-based commensality, *t*(94) = 7.19, *p* < 0.001, Cohen’s *d* = 0.74, or Mukbang-based commensality, *t*(94) = 3.49, *p* = 0.004, Cohen’s *d* = 0.36. Based on the results of expected pleasantness, an in-person commensality elicited a stronger preference for unhealthy foods than solitary eating, *t*(94) = 3.96, *p* < 0.001, Cohen’s *d* = 0.41, or cloud-based commensality, *t*(94) = 4.53, *p* < 0.001, Cohen’s *d* = 0.47. None of the other pairwise comparisons reached the significance level on either measure, all *t*s < 2.32, *p*s > 0.13.

## Discussion

In summary, three major findings have emerged from this study. First, the results revealed the belief of our participants that a cloud-based commensality could alleviate feelings of loneliness without compromising one’s health by enhancing food intake or choices of unhealthy foods. These results are in line with our first hypothesis (H1) and suggest that the cloud-based commensality can serve as an “alone-but-together” context to satisfy the needs of a person for autonomy and connectedness at the same time (Merdin-Uygur and Hesapci, 2018). On one hand, this type of social context can provide co-diners with an experience of being in the company of each other as well as opportunities for communication and interactions. Such social benefits are important due to the influence of social relationships on human health (Tay et al., 2013), especially when people are trying to maintain a long-distance relationship (Spence et al., 2019) or being forced to conduct daily activities online (Spence, 2020) in the period of going through a social lockdown due to the pandemic of the potentially deadly virus (Dimmock et al., 2021). On the other hand, an “alone-but-together” context is helpful if people have the desire to interact with others while eating together, but they also try to strategically avoid a social context, which often makes them eat more or choose less healthy foods than they ordinarily would. Eating together has been identified as such a context featured with social facilitation of eating, as it not only makes people eat more than usual (Cruwys et al., 2015) and provides an opportunity to overindulge (Herman, 2017) but also can make eating unhealthy foods even more pleasant (Huang et al., under review). It is possible that people may concentrate more on the social interaction rather than the foods or eating while engaging in the cloud-based commensality, which is different from the food-focused context of self-reflection or remote norms where social facilitation can be observed in the absence of others (Feeney et al., 2011; Nakata and Kawai, 2017).

Second, our results revealed that our participants expected their loneliness could be reduced when they were asked to imagine engaging in the Mukbang-based commensality, which is in line with the previous literature (Kircaburun et al., 2020). These results indicated that our participants were aware of the emotional benefits of the Mukbang-based commensality, although 42% of them did not engage in this type of eating. Our results also shed light on their reasons not to do so in daily life. For one thing, the Mukbang-based commensality was expected to be less effective than the cloud-based commensality in reducing loneliness, presumably as the cloud-based commensality could elicit the feeling of being accompanied by family or friends (Ceccaldi et al., 2020), not just by a broadcast jockey or other viewers as the Mukbang-based commensality did (Spence et al., 2019). For another, our participants also noticed that Mukbang-based commensality could increase one’s intake of food, decrease the probability of choosing healthy foods, and reduce the pleasantness of eating healthy foods. Numerous studies have documented the influence of social modeling on food intake (Cruwys et al., 2015), for example, eating while watching television may increase consumption of unhealthy foods (Marquis et al., 2002), and such harmfulness may be further aggravated by the overindulging Mukbang host or hostess (Donnar, 2017). Even though the food intake of an individual is usually immune to the eating behavior of a remote co-diner eating in a different context (Hermans et al., 2012), the situation in the Mukbang-based commensality might be different, as the host or hostess might provide a much more extreme social norm than ordinary co-diners would. That being said, our results revealed that the Mukbang-based commensality was expected to satisfy the need for privacy, which is very important for a solo diner in a very vulnerable state (Donnar, 2017). However, if Mukbang viewers try to obtain vicarious satisfaction of a desire to eat unhealthy foods and urge themselves to engage in healthy eating (Choe, 2019), our results suggest that the outcomes are more likely to be opposite to what they wish for.

Third, our results revealed that eating together was also expected to reduce loneliness and to enhance the pleasantness of eating unhealthy foods, which is in line with recent findings of Huang et al. (under review). Moreover, our results provide new empirical evidence that eating together could increase the probability of choosing unhealthy foods. These findings can be used to explain why a commensality is so desirable, but they also highlight the health-related risks of eating with others, such as the possibility of shifting an individual toward unhealthier food intake and choices (Cruwys et al., 2015; Herman, 2017). Taken together, our second and third findings are both in line with our second hypothesis (H2). By contrast, our results also revealed the belief of our participants in the advantages of solo dining, even though we mainly used this type of eating scenario as a baseline in this study. That is, our results suggest that eating without companions is helpful when an individual is mainly concerned about avoiding a social context facilitating overindulgence (Herman, 2017; Kircaburun et al., 2020), or focuses on resisting the temptation of eating unhealthy foods with others (Huang et al., under review). In other words, our results suggest that eating alone in a private situation can be used as a strategy to promote healthier eating (Moon et al., 2020). Moreover, it is important to differentiate choosing to eat alone from having to eat alone, as freely choosing to eat alone can reduce stress and make an individual feel relaxed (Nguyen et al., 2018), especially when a diner wants to have a quick meal or freely choose what to eat (Takeda and Melby, 2017).

As with any study, there are certain limitations as far as the interpretation and generalizability of this study are concerned. For one thing, we only tested healthy Chinese adults in this study, and they were undergraduate or graduate students when taking part in this study. However, individuals from different age groups or cultural backgrounds may have different beliefs in social eating (e.g., Cho et al., 2015; Wang et al., 2021). Therefore, cautions are called for if one tries to generalize our findings to populations of other age groups, other cultural backgrounds, or non-student samples (Henrich et al., 2010). Second, 68% of our participants indicated that they did not engage in the cloud-based commensality in daily life. Considering that the new pattern of at-home consumption of foods and eating experience brought by the COVID-19 pandemic (Plata et al., 2021; Spence et al., 2021), the present study revealed beliefs of people in the influence of the expected eating situation. Consequently, it will be necessary and interesting to conduct follow-up studies in which having participants actually eat and experience different types of digital commensality in order to test the influence of actual experience on their food choice and intake. Third, although we made the manipulation check on the food stimuli, we did not check whether the participants, indeed, identified with the person presented in the scenario image. Therefore, future research is needed to take this procedure into consideration.

## Conclusion

Our findings revealed the beliefs of people in both similarities and differences among in-person, Mukbang-based, and cloud-based commensalities in terms of the consequences on the emotional state, food intake, and food choice of a person. Compared to the unwanted social facilitation of eating in the traditional form of a commensality (Cruwys et al., 2015; Herman, 2017) or the risks of overindulging while watching Mukbang (Kircaburun et al., 2020), our results suggest that the cloud-based commensality can balance the need for social interactions with the strategic avoidance of a social context facilitating unhealthy eating. These findings shed light on the promising future of promoting the cloud-based commensality as an alternative to social gatherings if needed (Ceccaldi et al., 2020). Importantly, our findings characterize the cloud-based commensality as an efficient and adaptive approach to integrate the strengths of solitary and commensal eating *via* affordable and easy-to-implement technologies (i.e., smartphones), shedding light on how to use technologies to integrate social factors and food pleasure to promote healthy eating and to facilitate health self-management.

## Data Availability Statement

The raw data supporting the conclusions of this article will be made available by the authors, without undue reservation.

## Ethics Statement

The studies involving human participants were reviewed and approved by the Institution Review Board of Tsinghua University. The patients/participants provided their written informed consent to participate in this study.

## Author Contributions

XW and CW developed the idea for the study and drafted the manuscript. CW, YP, and XW collaboratively designed the study. CW and LQ collected the data. CW analyzed the data and conducted the interpretation of the data. YP and LQ provided critical revisions. All authors have approved the final version of the manuscript.

## Conflict of Interest

The authors declare that the research was conducted in the absence of any commercial or financial relationships that could be construed as a potential conflict of interest.

## Publisher’s Note

All claims expressed in this article are solely those of the authors and do not necessarily represent those of their affiliated organizations, or those of the publisher, the editors and the reviewers. Any product that may be evaluated in this article, or claim that may be made by its manufacturer, is not guaranteed or endorsed by the publisher.
